# Effects of Electronic Nicotine Delivery Systems Substitution on Body Weight Status: Protocol for a Systematic Review and Meta-Analysis

**DOI:** 10.2196/56324

**Published:** 2024-03-26

**Authors:** Giusy Rita Maria La Rosa, Maria Ahmed Qureshi, Lucia Frittitta, Erika Anastasi, Riccardo Polosa

**Affiliations:** 1 Department of Clinical and Experimental Medicine University of Catania Catania Italy; 2 Centre of Excellence for the Acceleration of Harm Reduction University of Catania Catania Italy; 3 Diabetes and Obesity Center Garibaldi Hospital Catania Italy; 4 ECLAT Srl Spin-off of the University of Catania Catania Italy; 5 Centre for the Prevention and Treatment of Tobacco Addiction University Hospital University of Catania Catania Italy

**Keywords:** Electronic Nicotine Delivery Systems, body weight, smoking cessation, tobacco harm reduction, systematic review, meta-analysis

## Abstract

**Background:**

Weight gain following smoking cessation is a well-documented concern, often attributed to the absence of nicotine’s metabolic influence. The adoption of Electronic Nicotine Delivery Systems (ENDS) has been used to achieve smoking cessation, with claims of aiding weight control. However, existing reviews present conflicting conclusions on ENDS’ impact on weight status, necessitating a rigorous evaluation.

**Objective:**

We aim to conduct a systematic review with meta-analysis to assess the actual impact of ENDS on weight status in individuals who have ceased or reduced conventional smoking. The primary goal is to provide clinicians with evidence-based insights into the potential effects of ENDS use as a smoking substitute on weight control.

**Methods:**

Adhering to PRISMA-P (Preferred Reporting Items for Systematic Reviews and Meta-Analyses Protocols) guidelines, our systematic review will analyze randomized and nonrandomized controlled trials, clinical trials (quasi-experimental), and prospective or retrospective cohort studies on the weight status effects of ENDS among individuals who have either quit or reduced smoking. Searches will include PubMed, Scopus, and Cochrane Library, covering the period from 2010 to January 2024. A gray literature search and supplementary searches will be performed. Data will be extracted independently by 2 reviewers and quality assessments will be conducted concurrently. Quality assessments will use Joanna Briggs Institute tools, 2020 version, along with bias assessments for internal validity and reporting bias based on the Catalogue of Bias. The included studies will be examined for any internal data reporting discrepancies by using Puljak’s checklist. Meta-analysis and subgroup analyses (ie, general ENDS usage, ENDS use coupled with a reduction in smoking exceeding 50%, and exclusive ENDS use for achieving smoking cessation) are planned. Certainty of evidence will be evaluated using the Grading of Recommendations Assessment, Development, and Evaluation (GRADE) framework.

**Results:**

The protocol has been registered in PROSPERO (CRD42023494974) and the entire systematic review is expected to be completed by April 2024. The main goal of this review is to retrieve all current human research studies investigating the influence of ENDS on weight management among individuals who have quit or reduced smoking. Furthermore, the review will assess the quality of these studies and examine potential biases to identify the most dependable evidence available. Dissemination strategies will include traditional journal publications, social media announcements, and a white paper. The latter, available for download and distributed at conferences, aims to reach a broad audience, including clinicians and ENDS users.

**Conclusions:**

The review will address the importance of informing health care professionals and patients about the current and robust evidence regarding the effects of transitioning to ENDS for smoking cessation on weight status.

**Trial Registration:**

PROSPERO CRD42023494974; https://www.crd.york.ac.uk/prospero/display_record.php?RecordID=494974

**International Registered Report Identifier (IRRID):**

PRR1-10.2196/56324

## Introduction

### Background

The phenomenon of weight gain following smoking cessation is a well-documented concern in scientific research [1]. Typically, individuals who quit smoking experience an average weight increase ranging from 4.67 to 4.8 kg after 1 year, with more than 10% experiencing a rise exceeding 10 kg [2,3]. This weight gain can primarily be attributed to the lack of nicotine’s influence on metabolic processes, which includes appetite suppression and alterations to both lipolysis and lipogenesis [4,5].

In the past few years, there has been a notable increase in the adoption of Electronic Nicotine Delivery Systems (ENDS), commonly known as e-cigarettes, among individuals attempting to cease tobacco use [6]. Some who use ENDS do so for weight loss or weight management. The use of ENDS for weight loss was most prevalent among individuals with eating disorders, at 32% (n=178) [7]. This usage drops considerably in the general ENDS-using population. In the United States, 15% (13/84) of college students and 13.5% (62/459) of adults reported using ENDS for weight control [8,9]. Among US military recruits, 5.1% (11/223) of males and 6.0% (4/61) of females have used ENDS for this purpose [10]. In England, the figure stands at 4.6% (18/394) of current ENDS users [3].

Observational studies have also noted the practice of using vaping as a dietary substitution, where individuals vape flavored liquids to mitigate food cravings. This behavior, while less prevalent in England [3], has been reported in various surveys and interviews conducted in the United States [11,12].

Commercial entities have recognized these consumer patterns, resulting in the development and marketing of vaping products that claim to support weight loss. Numerous patents have been filed globally, and several companies have marketed vapor devices that propose to offer benefits such as fat burning, appetite suppression, and rapid weight loss [13,14]. These claims, often promoted through online sites and aggressive marketing strategies [13,15], are claims that have not been verified.

### Previous Reviews

Recent reviews assert that there is insufficient evidence to draw definitive conclusions on the impact of ENDS on weight control in people who have ceased or reduced conventional smoking. The systematic review of Hod et al [16] of human and in vitro studies concluded that ENDS use is prevalent among the obese, although the authors state that causality cannot be determined due to the cross-sectional design of human studies. Moreover, the data are conflicting, with in vivo studies suggesting weight loss effects, but in vitro studies do not support this claim Thus, the authors underlined the need for further investigation to determine the impact of ENDS on weight control [16]. Another review by Hartmann-Boyce et al [17] found 2 ENDS studies, 1 involving ENDS as an adjunct to nicotine replacement therapy and the other comparing them to varenicline, both with wide CIs at all measurement time points. The CIs of weight change (kg) at the end of the treatment and 12 months encompassed both clinically significant weight loss and weight gain [17]. Therefore, the authors stated that “data are needed on whether using e-cigarettes to quit smoking affects post-cessation weight change” [17].

### Research Question

Given the rise of ENDS as an alternative to traditional smoking, and their associated perceived benefits for weight control, we aim to rigorously evaluate the actual impact of ENDS on weight status in individuals who have ceased or reduced conventional smoking. For this purpose, we intend to conduct a systematic review with meta-analysis to analyze human clinical studies that provide longitudinal data on body weight among participants who have replaced cigarette smoking with ENDS use.

### Population, Intervention, Comparator, and Outcomes Criteria

The research strategy follows the Population, Intervention, Comparator, and Outcome (PICO) approach as follows: population—adults who smoke cigarettes, intervention—complete or partial substitution of ENDS for cigarettes, comparator—within-subject changes baseline to the end of the study, and outcomes—changes in body weight.

### Objectives

We will conduct a systematic review with a meta-analysis to critically assess and synthesize available human studies on the weight status effects of ENDS among individuals who have either quit or reduced smoking. Our goal is to critically evaluate and synthesize the available evidence to offer clinicians high-quality insights into the potential effects of ENDS use as a substitute for smoking, specifically concerning weight control. Thus, our aim is to equip clinicians with robust evidence to enhance their treatment recommendations and strategies for individuals who smoke, aiding them in making informed decisions and plans.

## Methods

### Overview

In adherence to the PRISMA-P (Preferred Reporting Items for Systematic Reviews and Meta-Analyses Protocols) requirements [18], this protocol is registered with PROSPERO (CRD42023494974), and any deviations will be transparently reported within the review. The review team (GRMLR, MAQ, LF, EA, and RP), led by RP, has substantial expertise in conducting literature reviews and an expert background in tobacco control, tobacco harm reduction, and ENDS.

### Database Search and Secondary Searches

The databases to be included are PubMed, Scopus, and CENTRAL Cochrane Library. Using the keywords specific to each database (ie, weight, BMI, “body mass index,” “electronic cigarette,” “e-cigarette,” and “electronic nicotine”), searches will be performed in the title and abstract fields in PubMed, and in the title, abstract, and keywords in Scopus. Cochrane Library will be searched for trials. The search will include the period from 2010 to January 2024, in line with the date of the first peer-reviewed research studies on ENDS. Language restrictions will not be applied. In compliance with PRISMA-P, the search strategy example is detailed in Multimedia Appendix 1. Retrievals will be downloaded into EndNote (Clarivate), and duplicates will be removed.

A secondary search of the existing reviews, conducted independently by 2 reviewers, will be conducted to identify any additional studies. Next, the references of the included studies will be scrutinized to identify additional studies. Finally, the included studies will be citation chased (ie, snowball search) through Google Scholar. The studies excluded during the thorough full-paper review will be documented.

The list of selected studies will be forwarded to 2 medical experts to validate that all pertinent studies have been included. In addition, a comprehensive gray literature search will be conducted on the websites of 16 weight-related medical organizations (Multimedia Appendix 1).

### Inclusion and Exclusion Criteria

The selected study designs will include randomized and nonrandomized controlled trials, clinical trials (quasi-experimental), and prospective or retrospective cohort studies. The initial exclusion will be based on titles and abstracts reviewed. Exclusion criteria will be by publication types (ie, editorials, letters, commentaries, protocols, narrative reviews, conference abstracts, and dissertations), study designs (ie, vitro, animal studies, inhalation toxicology, biomarker studies, surveys, and qualitative studies), adolescent populations, and studies lacking baseline or follow-up weight data. A total of 2 reviewers will independently conduct the exclusion process, with any discrepancies resolved through discussion.

The next process will involve a thorough review of full papers, guided by 3 inclusion criteria, including study designs, availability of data before and after testing participants who substituted ENDS for smoking, and outcome data on weight. All 3 criteria will have to be satisfied for a study to be included.

Review team members will undergo training on the inclusion criteria through analysis of 3 included studies. Two independent reviewers will then conduct the inclusion and exclusion assessments on the studies, resolving any disparities through discussion. The objective is to attain a high level of agreement between reviewers on the study selection process. The project leader will make the final decision regarding the inclusion and assessment of the studies.

### Data Extraction

Using a standardized form derived from Joanna Briggs Institute (JBI) and Cochrane Collaboration inventories [19,20], data extraction will be conducted by 2 reviewers independently, followed by cross-checking for accuracy. The full data extraction form is provided in Multimedia Appendix 1. The data extraction form includes bibliographic details, study population demographics, intervention descriptions, and weight measurements. The form will be pilot-tested and revised as necessary.

In cases where the published data are insufficient or absent, an email requesting additional details will be sent to the corresponding author. Findings from the included studies will be reported in a dual format. First, individual studies will be summarized with a concise narrative description. Second, study tables will be formulated incorporating elements derived from the data extraction and quality assessments (refer to the subsequent section).

### Quality Assessment and Risk of Bias

Quality assessments will use JBI tools, 2020 version [21], along with bias assessments for internal validity and reporting bias based on the Catalogue of Bias [22] (Multimedia Appendix 1). Examination of studies for any internal data reporting discrepancies will be conducted using the checklist proposed by Puljak et al [23] (Multimedia Appendix 1). The data extraction forms will be pilot-tested and revised if necessary. The comprehensive evaluation of study quality will involve calculating an overall rating through a rubric that integrates the JBI score and biases checklist. The overall study quality will be categorized according to the Cochrane guidelines—low risk of bias, some concerns, and high risk of bias [20]. Quality assessment will be conducted concurrent with the data extraction, and 2 reviewers will independently perform assessments. Discrepancies will be resolved through discussion or decided by the project leader.

No studies will be excluded on the basis of their quality assessment or bias. Studies deviating from the JBI quality assessment criteria or exhibiting biases will be documented in the study table. These limitations will be explicitly identified during the data analysis and referenced in the discussion section of the review.

### Data Analysis and Synthesis

Studies will be categorized by risk of bias, and summaries will include study design, participant and intervention characteristics, and main findings. A meta-analysis of change scores is planned, where the change score is defined as mean kg at the end of the study minus mean kg at the baseline. Three subgroup analyses will be performed, focusing on (1) general ENDS usage, (2) ENDS use coupled with a reduction in smoking exceeding 50%, and (3) exclusive ENDS use with the goal of smoking cessation. Publication bias will be assessed if a minimum of 10 studies are included.

### Grading of Recommendations Assessment, Development, and Evaluation

Certainty of evidence will be evaluated using the Grading of Recommendations Assessment, Development, and Evaluation (GRADE) framework [24].

## Results

The protocol has been registered in PROSPERO (CRD42023494974) and the entire systematic review is expected to be completed by April 2024. The primary objective of this review is to compile all existing human research studies concerning the impact of ENDS on weight control in people who ceased or reduced smoking. In addition, the review will rigorously evaluate the quality of these studies and scrutinize potential biases to emphasize the most reliable and available evidence. The planned review will also identify studies that may exhibit reporting bias, thereby enabling a more accurate representation of the effects of ENDS on weight control within the ongoing discourse on tobacco harm reduction.

## Discussion

### Principal Findings

This protocol is intended to provide the methodological framework for a systematic review and meta-analysis of the actual impact of ENDS on weight status in individuals who have ceased or reduced conventional smoking.

Many research protocols lack comprehensive planning for dissemination and knowledge translation activities beyond the conventional approach of publishing the review in a peer-reviewed journal and presenting findings at conferences. Naturally, this review will be disseminated through these traditional channels. Following an approach previously described [25], some additional strategies will be adopted. Briefly, upon acceptance, the project protocol will be published in JMIR Research Protocols and the final review with meta-analysis will be published in a peer-reviewed journal. In addition, efforts will be made to translate the review’s abstract into several languages. To enhance visibility, the availability of the review will be disseminated via social media platforms.

A white paper will be produced and made available for download on a dedicated website, and we anticipate disseminating it to medical associations and distributing it at conferences. The goal is to ensure the widespread of findings among clinicians and individuals currently using or considering ENDS. Given that a significant number of physicians and health care providers harbor misconceptions regarding the health impacts of nicotine [26,27], the white paper will incorporate a dedicated section focusing on nicotine. Infographics drawn from the white paper will be disseminated to current and potential ENDS users via user advocacy organizations [25]. Traditional review publication methods may not effectively reach the stakeholders who will benefit from clear and accessible updated sources of information. The objective of these activities is to ensure broad dissemination of the review findings to multiple audiences.

It is crucial to acknowledge the potential limitations arising from both the number and the quality of the studies that will be included in this systematic review. The limited quantity of available studies can restrict the scope and depth of conclusions drawn, leading to gaps in understanding and potentially biasing the overall assessment. Furthermore, the quality of the studies significantly impacts the reliability of the review’s conclusions. If the included studies exhibit methodological flaws, biases, or insufficient rigor, the overall evidence may be weakened, resulting in tentative or inconclusive findings.

### Conclusions

In conclusion, tobacco consumption constitutes a significant public health burden. Electronic cigarettes may represent a viable option for those attempting to quit smoking. Furthermore, there is a growing interest in these devices due to their potential benefits for weight control, especially for counteracting the short-term weight gain associated with smoking cessation. Therefore, it is essential to inform both patients and health care professionals about the most current and robust evidence regarding the effects of switching to electronic cigarettes for smoking cessation on weight status. This review, including meta-analysis, aims to contribute to this objective.

## Appendix

Multimedia Appendix 1 (A) Sample PubMed bibliographic search. (B) Weight medical organizations grey literature search. (C) Data extraction form. (D) Data discrepancies form. (E) Bias report. (F) PRISMA-P 2015 checklist.

## Abbreviations

ENDS: electronic nicotine delivery systems
GRADE: Grading of Recommendations Assessment, Development, and Evaluation
JBI: Joanna Briggs Institute
PICO: Population, Intervention, Comparator, and Outcome
PRISMA-P: Preferred Reporting Items for Systematic Reviews and Meta-Analyses Protocols

## Data Availability

Data sharing is not applicable to this study as no data sets were generated or analyzed during this study.
