# Supplementary material for: Effects of Electronic Nicotine Delivery Systems Substitution on Body Weight Status: Protocol for a Systematic Review and Meta-Analysis
Source: JMIR Res Protoc. 2024 Mar 26;13:e56324. doi: 10.2196/56324 (PMC11005425; doi:10.2196/56324)
Supplement: Multimedia Appendix 1 [file resprot_v13i1e56324_app1.docx]

### A. Sample PubMed bibliographic search………………………………………...2

### B. Weight medical organizations grey literature search…………………….........3

### C. Data extraction form……………………………………………...…………...4

### D. Data discrepancies form…………………………………………..…………...6

### E. Bias report…………………………………………………………...…………7

### F. PRISMA-P 2015 Checklist…………………………………………………….8

### A. Sample PubMed bibliographic search

("smoking"[MeSH Terms] OR "smoking"[Title/Abstract] OR "smok*"[Title/Abstract] OR "cigarette"[Title/Abstract]) AND ("cessation"[Title/Abstract] OR "quit*"[Title/Abstract] OR "abstinence"[Title/Abstract] OR "stop*"[Title/Abstract] OR "e-cigarett*"[Title/Abstract] OR "electronic cigarette" [Title/Abstract] OR "Electronic Nicotine Delivery Systems"[MeSH Terms] OR "ENDS" [Title/Abstract] OR "electronic nicotine" [Title/Abstract] OR "vaping" [MeSH Terms] OR "vaping" [Title/Abstract]) AND ("Body Weight"[MeSH Terms] OR "Body Weight"[ Title/Abstract] OR "Body Weight Changes"[MeSH Terms] OR "Body Weight Changes"[Title/Abstract] OR "obesity"[MeSH Terms] OR "obesity"[Title/Abstract] OR "weight control" [Title/Abstract] OR "Weight Loss"[MeSH Terms] OR "Weight Loss"[Title/Abstract] OR "Body Mass Index"[MeSH Terms] OR "Body Mass Index"[Title/Abstract] OR

"BMI"[Title/Abstract])

### B. Weight medical organizations grey literature search

| American Association of Clinical Endocrinology (AACE) | https://www.aace.com/ |
| --- | --- |
| American Board of Obesity Medicine (ABOM) | https://www.abom.org/ |
| American Society for Metabolic and Bariatric Surgery (ASMBS) | https://asmbs.org/ |
| Asia Oceania Association for the Study of Obesity (AOASO) | http://aoaso.org/ |
| Australian and New Zealand Obesity Society (ANZOS) | https://www.anzos.com/ |
| Columbia Endocrinology Metabolic and Weight Control Center | https://www.columbiaendocrinology.com/ |
| Comprehensive Weight Control Center at Weill Cornell Medicine | https://weillcornell.org/ |
| International Federation for the Surgery of Obesity and Metabolic Disorders (IFSO) | https://www.ifso.com/ |
| Endocrine Society | https://www.endocrine.org/ |
| European Association for the Study of Obesity (EASO) | https://easo.org/ |
| Mayo Clinic Advanced Weight Management Program | https://www.mayoclinic.org/ |
| Obesity Canada | https://obesitycanada.ca/ |
| Obesity Medicine Association (OMA) | https://obesitymedicine.org/ |
| The Obesity Society (TOS) | https://www.obesity.org/ |
| University of Michigan Weight Management & Obesity Program | https://www.uofmhealth.org/ |
| World Obesity Federation | https://www.worldobesity.org/ |

### C. Data extraction form

Reviewer: Date: Verified on:

Title:

Year:

Journal: DOI: PMID:

1st Author:

COI Declaration:

2nd Author

COI Declarations:

[all additional authors]

Additional/supplementary files [identify or NONE]

Published protocol or trial registry [identify or NONE]

Funder:

Research question (or goal if no stated question)

Study design:

Location (Country):

Setting:

Participants:

Age

Sex

Exclusion criteria

Smoking status/history/current use

Other tobacco products reported

ENDS history

Disease status

Other descriptors (economic status, etc.)

Intervention

ENDS device and liquid

ENDS training

Frequency of use

Duration of exposure

Concurrent use of tobacco

Follow-up periods

Drop-outs

Fidelity including relapse to cigarette use during study

Tests/data

Named tests

Testing protocols including pretest abstinence period

Observational data (disease symptoms)

Self-report data

Verification of tobacco abstinence

Tests results/data [for FINAL follow-up period] used in review analysis

Dual user and exclusive users reported separately or combined

Secondary outcomes (tobacco cessation, ENDS cessation, tobacco relapse)

Statistical tests

Statistical analyses

Limitations identified by authors

Recommendations for future research

Conclusions(s) quotations (with page number)

Reviewer Comments:

:

### D. Data discrepancies form

Reviewer: Date: Verified on:

Author (year):

Indicate status:

No discrepancies observed.

Discrepancies in data between the abstract and the study text:

Discrepancies within the text (compare all references in the text to the data):

Discrepancies between the text and figure:

Discrepancies between the text and table:

Discrepancies in number of participants:

Study corresponding author contacted on date and text of email:

(If no reply): Other authors and cc of journal editor on date and text of email:

### E. Bias report

Reviewer date verification

Author (year)

Reporting Biases

Spin Bias

Data-dredging bias

Hypothetical bias

All’s well literature bias

Ascertainment bias

Biases of rhetoric

Compliance bias

Confirmation bias

Detection bias

Hot stuff bias

Industry Sponsorship Bias

Misclassification bias

One-sided reference bias

Partial reference bias

Performance bias

Popularity bias

Prevalence-incidence (Neyman) bias

Selection bias

Substitution game bias

Volunteer bias

Wrong sample size bias

### **F. PRISMA-P 2015 Checklist**

# **This checklist has been adapted for use with protocol submissions to *Systematic Reviews* from Table 3 in Moher D et al:** Preferred reporting items for systematic review and meta-analysis protocols (PRISMA-P) 2015 statement. *Systematic Reviews* 2015 **4**:1

| **Section/topic** | **#** | **Checklist item** | **Information reported** | | **Page number** |
| --- | --- | --- | --- | --- | --- |
|  |  |  | **Yes** | **No** |  |
| **ADMINISTRATIVE INFORMATION** | | | | | |
| **Title** | | | | | |
| Identification | 1a | Identify the report as a protocol of a systematic review | X |  | 1 |
| Update | 1b | If the protocol is for an update of a previous systematic review, identify as such |  |  | Not applicable |
| **Registration** | 2 | If registered, provide the name of the registry (e.g., PROSPERO) and registration number in the Abstract | X |  | 2,3,6 |
| **Authors** | | | | | |
| Contact | 3a | Provide name, institutional affiliation, and e-mail address of all protocol authors; provide physical mailing address of corresponding author | X |  | 1 |
| Contributions | 3b | Describe contributions of protocol authors and identify the guarantor of the review | X |  | 11 |
| **Amendments** | 4 | If the protocol represents an amendment of a previously completed or published protocol, identify as such and list changes; otherwise, state plan for documenting important protocol amendments |  |  | Not applicable |
| **Support** | | | | | |
| Sources | 5a | Indicate sources of financial or other support for the review | X |  | 11 |
| Sponsor | 5b | Provide name for the review funder and/or sponsor | X |  | 11 |
| Role of sponsor/funder | 5c | Describe roles of funder(s), sponsor(s), and/or institution(s), if any, in developing the protocol | X |  | 11 |
| **INTRODUCTION** | | | | | |
| **Rationale** | 6 | Describe the rationale for the review in the context of what is already known | X |  | 4,5 |
| **Objectives** | 7 | Provide an explicit statement of the question(s) the review will address with reference to participants, interventions, comparators, and outcomes (PICO) | X |  | 6 |
| **METHODS** | | | | | |
| **Eligibility criteria** | 8 | Specify the study characteristics (e.g., PICO, study design, setting, time frame) and report characteristics (e.g., years considered, language, publication status) to be used as criteria for eligibility for the review | X |  | 7,8 |
| **Information sources** | 9 | Describe all intended information sources (e.g., electronic databases, contact with study authors, trial registers, or other grey literature sources) with planned dates of coverage | X |  | 6,7 |
| **Search strategy** | 10 | Present draft of search strategy to be used for at least one electronic database, including planned limits, such that it could be repeated | X |  | Suppl. Materials |
| ***STUDY RECORDS*** | | | | | |
| Data management | 11a | Describe the mechanism(s) that will be used to manage records and data throughout the review | X |  | 7 |
| Selection process | 11b | State the process that will be used for selecting studies (e.g., two independent reviewers) through each phase of the review (i.e., screening, eligibility, and inclusion in meta-analysis) | X |  | 8 |
| Data collection process | 11c | Describe planned method of extracting data from reports (e.g., piloting forms, done independently, in duplicate), any processes for obtaining and confirming data from investigators | X |  | 8, Suppl. Materials |
| **Data items** | 12 | List and define all variables for which data will be sought (e.g., PICO items, funding sources), any pre-planned data assumptions and simplifications | X |  | 8, Suppl. Materials |
| **Outcomes and prioritization** | 13 | List and define all outcomes for which data will be sought, including prioritization of main and additional outcomes, with rationale | X |  | 6, Suppl. Materials |
| **Risk of bias in individual studies** | 14 | Describe anticipated methods for assessing risk of bias of individual studies, including whether this will be done at the outcome or study level, or both; state how this information will be used in data synthesis | X |  | 8,9, Suppl. Materials |
| ***DATA*** | | | | | |
| **Synthesis** | 15a | Describe criteria under which study data will be quantitatively synthesized | X |  | 9 |
|  | 15b | If data are appropriate for quantitative synthesis, describe planned summary measures, methods of handling data, and methods of combining data from studies, including any planned exploration of consistency (e.g., *I* ^2^, Kendall’s tau) | X |  | 9 |
|  | 15c | Describe any proposed additional analyses (e.g., sensitivity or subgroup analyses, meta-regression) | X |  | 9 |
|  | 15d | If quantitative synthesis is not appropriate, describe the type of summary planned | X |  | 9 |
| **Meta-bias(es)** | 16 | Specify any planned assessment of meta-bias(es) (e.g., publication bias across studies, selective reporting within studies) | X |  | 9 |
| **Confidence in cumulative evidence** | 17 | Describe how the strength of the body of evidence will be assessed (e.g., GRADE) | X |  | 9 |
